# Evaluation of coronary flow is useful in patients with left coronary cusp thrombus formation after left ventricular assist device implantation

**DOI:** 10.1093/ehjcr/ytad025

**Published:** 2023-01-12

**Authors:** Tasuku Sato, Michinari Hieda, Shutaro Futami, Mitsuhiro Fukata, Akira Shiose

**Affiliations:** Heart Center, Kyushu University Hospital, Fukuoka, Japan; Department of Medicine and Bio-systemic Science, Hematology, Oncology, and Cardiovascular Medicine, Kyushu University Hospital, School of Medicine, Kyushu University, 3-1-1 Maidashi Higashi-Ku Fukuoka City, Clinical Research Building B7F, Fukuoka 812-8586, Japan; Department of Medicine and Bio-systemic Science, Hematology, Oncology, and Cardiovascular Medicine, Kyushu University Hospital, School of Medicine, Kyushu University, 3-1-1 Maidashi Higashi-Ku Fukuoka City, Clinical Research Building B7F, Fukuoka 812-8586, Japan; Department of Medicine and Bio-systemic Science, Hematology, Oncology, and Cardiovascular Medicine, Kyushu University Hospital, School of Medicine, Kyushu University, 3-1-1 Maidashi Higashi-Ku Fukuoka City, Clinical Research Building B7F, Fukuoka 812-8586, Japan; Department of Cardiovascular Surgery, Kyushu University Hospital, Fukuoka, Japan

## Abstract

Left ventricular assist device (LVAD) is essential for patients with severe heart failure, but there is a risk of thrombus formation on the aortic root and cusps, leading to coronary artery occlusion. Even with the narrowing of the echo-window due to LVAD, careful observation of coronary flow by transthoracic echocardiography can evaluate the patency of coronary flow non-invasively and immediately.

## Case description

A 19-year-old man with advanced heart failure (idiopathic dilated cardiomyopathy) underwent left ventricular assist device (LVAD; HeartMate 3) implantation and aortic valve closure (Park’s stitch). On postoperative Day 8, transthoracic echocardiography (TTE) revealed a thrombus at the left coronary cusp (LCC) (*Panel A*). Coagulation functions, including Protein C, S, and plasminogen activator inhibitor 1, were within normal limits. The electrocardiogram showed sinus rhythm but had noise related to LVAD (*Panel B*). Therefore, the coronary arteries were delineated in the parasternal short-axis section to confirm the coronary flow of the left anterior descending (LAD). Optimization of settings for detecting the low-velocity flow of LAD was that (i) increased colour-Doppler gain, (ii) decreased colour-scale (Nyquist limit: 20 cm/s), and (iii) colour-Doppler dominance in tissue-priority adjustment. Coronary flow from the LAD ostium to the distal was identified. Thus, we confirmed that the thrombus did not cause LAD occlusion (*Panels C* and *D*; Supplementary material online, *Video S1*). Contrast-enhanced computed tomography showed LCC thrombus (*Panel E*) but no left coronary artery occlusion (*Panel F*; Supplementary material online, *Video S2*). Postoperatively, the patient was warfarinized with a PT-INR of 2.0–3.0. Fortunately, the thrombus disappeared on postoperative Day 35 (*Panel G*). Observing the aortic valve cusps and evaluating coronary flow with TTE during the perioperative period of LVAD implantation with a Park’s stitch is paramount. Even with the narrowing of the echo-window due to LVAD, careful TTE screening with setting optimization can help to evaluate coronary cusp thrombosis related to LVAD, preventing thrombotic adverse events.

**Figure:**
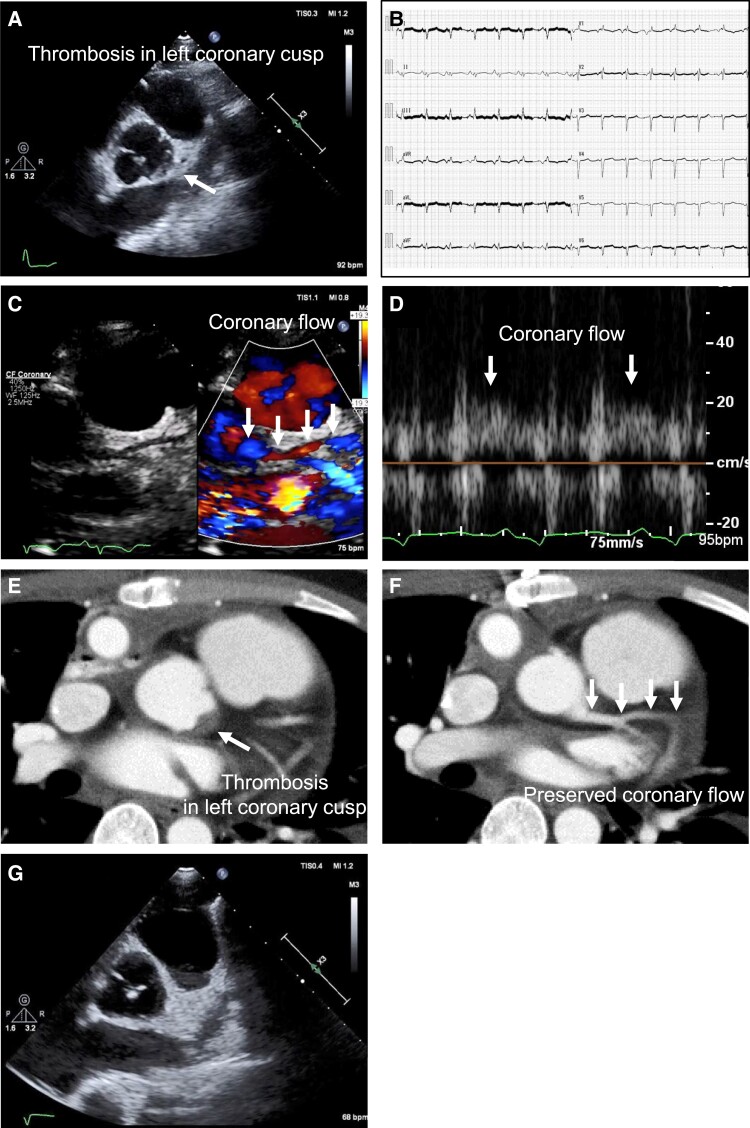


## Supplementary material

Supplementary material is available at *European Heart Journal – Case Reports*.

## Supplementary Material

ytad025_Supplementary_Data

## Data Availability

All data are incorporated into the article and its online Supplementary material.

