# Supplementary material for: Evaluation of coronary flow is useful in patients with left coronary cusp thrombus formation after left ventricular assist device implantation
Source: Eur Heart J Case Rep. 2023 Jan 12;7(1):ytad025. doi: 10.1093/ehjcr/ytad025 (PMC9883730; doi:10.1093/ehjcr/ytad025)
Supplement: ytad025_Supplementary_Data [file ytad025_supplementary_data.zip › Data Availability Statements.docx]

Data Availability Statements

**Availability of data**

All data are incorporated into the article and its online supplementary material.

**Sample statement**

The data underlying this article are available in the article and in its online supplementary material.
