# Supplementary material for: Evaluation of coronary flow is useful in patients with left coronary cusp thrombus formation after left ventricular assist device implantation
Source: Eur Heart J Case Rep. 2023 Jan 12;7(1):ytad025. doi: 10.1093/ehjcr/ytad025 (PMC9883730; doi:10.1093/ehjcr/ytad025)
Supplement: ytad025_Supplementary_Data [file ytad025_supplementary_data.zip › figure_EHJ-CR_Ver1.pptx]

## Slide 1
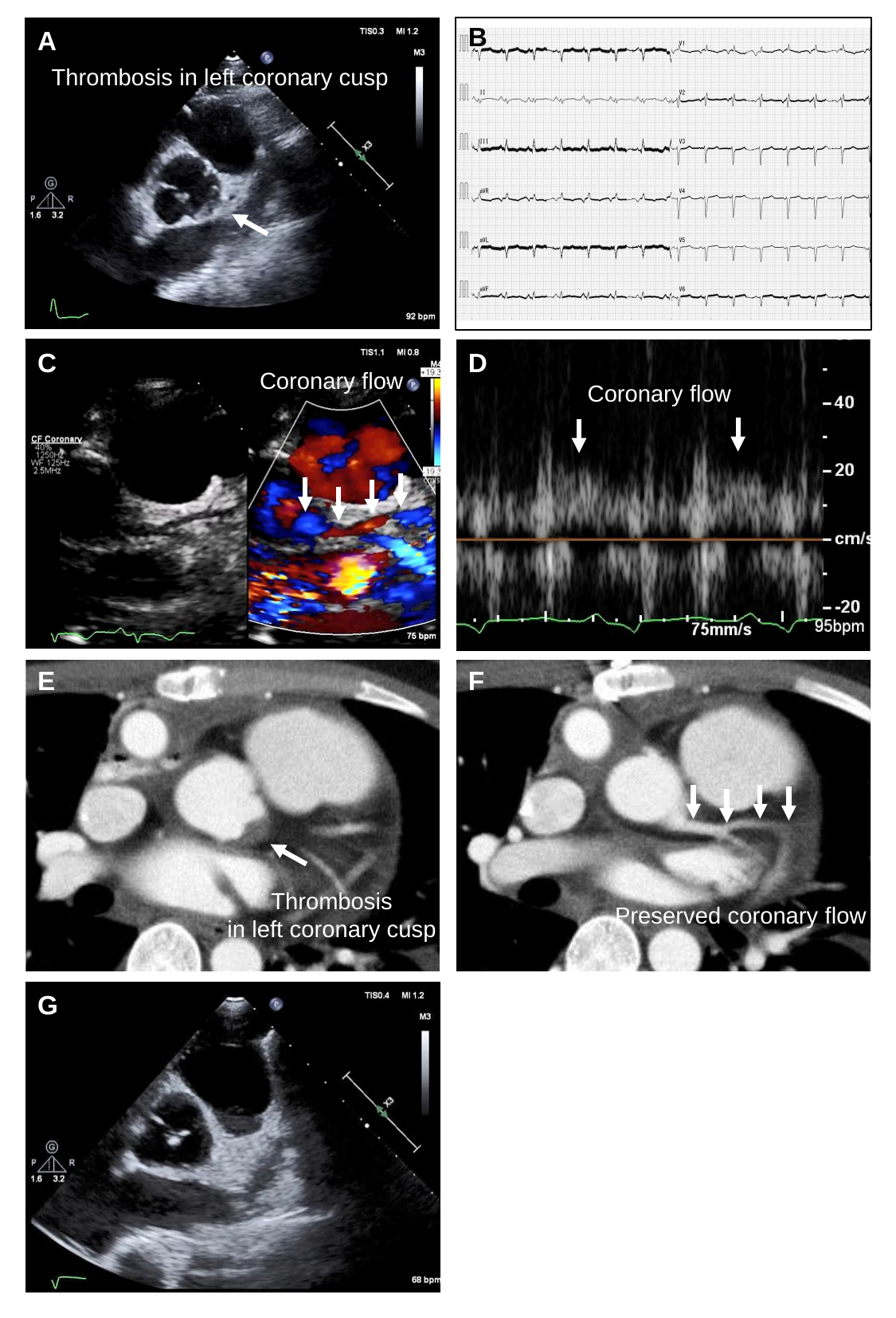

A
B
Thrombosis in left coronary cusp
C
Coronary flow
D
Coronary flow
E
F
Preserved coronary flow
Thrombosis
in left coronary cusp
G
